# Plasma Extracellular Vesicles Contain Protein Biomarkers for Capturing Stages of Metabolic Dysfunction-Associated Steatotic Liver Disease: A Preliminary Exploratory Study

**DOI:** 10.3390/biom15111596

**Published:** 2025-11-14

**Authors:** Yakun Li, Koen C. van Son, Sandra Serna-Salas, Justina C. Wolters, Nienke P. M. Wassenaar, Stan Driessen, Anne Linde Mak, Anne-Marieke van Dijk, Veera A. T. Houttu, Julia J. Witjes, Diona Zwirs, Michail Doukas, Joanne Verheij, Robin P. F. Dullaart, Hans Blokzijl, Adriaan G. Holleboom, Han Moshage

**Affiliations:** 1 Department of Gastroenterology and Hepatology, University Medical Center Groningen, University of Groningen, Hanzeplein 1, 9700 RB Groningen, The Netherlands; 2 Department of Vascular Medicine, Amsterdam UMC, University of Amsterdam, Meibergdreef 9, 1105 AZ Amsterdam, The Netherlands; 3 Department of Gastroenterology and Hepatology, Amsterdam UMC, University of Amsterdam, Meibergdreef 9, 1005 AZ Amsterdam, The Netherlands; 4 Amsterdam Gastroenterology Endocrinology Metabolism (AGEM) Institute, Amsterdam UMC, University of Amsterdam, Meibergdreef 9, 1105 AZ Amsterdam, The Netherlands; 5 Department of Paediatrics, University Medical Center Groningen, University of Groningen, Hanzeplein 1, 9700 RB Groningen, The Netherlands; 6 Department of Radiology and Nuclear Medicine, Amsterdam UMC, University of Amsterdam, Meibergdreef 9, 1105 AZ Amsterdam, The Netherlands; 7 Department of Pathology, Erasmus University Medical Center, Erasmus University Rotterdam, Molewaterplein 40, 3015 CN Rotterdam, The Netherlands; 8 Department of Pathology, Amsterdam UMC, University of Amsterdam, Meibergdreef 9, 1105 AZ Amsterdam, The Netherlands; 9 Department of Internal Medicine, Division of Endocrinology, University Medical Center Groningen, University of Groningen, Hanzeplein 1, 9700 RB Groningen, The Netherlands

**Keywords:** extracellular vesicles, metabolic dysfunction-associated steatotic liver disease, fibrosis, steatosis, steatohepatitis

## Abstract

Metabolic dysfunction-associated steatotic liver disease (MASLD) is increasing in both prevalence and severity, highlighting the need for non-invasive biomarkers to assess disease activity. Extracellular vesicles (EVs), which carry molecular cargo from their cells of origin, hold promise as accessible biomarkers. We performed proteomic profiling of plasma-derived EVs from 70 patients with MASLD to identify protein signatures associated with key histological features (steatosis, metabolic dysfunction-associated steatohepatitis (MASH), and fibrosis). These proteins were subsequently correlated with magnetic resonance (MR)-based liver imaging. Plasma EV protein profile differed between mild (S1) and advanced steatosis (S3). H4C1, OIT3, and ANPEP were elevated in S3, while CCDC25 and KLHL41 were decreased (|log_2_ fold change| > 1, *p* < 0.05). KLHL41 had a weak-to-moderate correlation with proton density fat fraction (PDFF) (R = −0.34, *p* = 0.016). GP1BA was upregulated in MASH (log_2_ fold change = 1.13, *p* = 0.03) but showed weak correlation with cT1, an imaging parameter for steatohepatitis (R = 0.22, *p* = 0.173). In fibrosis, complement component 7 (C7) was elevated in advanced (≥F3) vs. mild fibrosis (<F2) (log_2_ fold change = 0.95, adjusted *p* = 0.002) and correlated with MR elastography-derived liver stiffness (R = 0.38, *p* = 0.004). The AUC of C7 for differentiating <F2 vs. ≥F2 and <F3 vs. ≥F3 was 0.80 (95% CI: 0.69–0.91) and 0.83 (95% CI: 0.72–0.93), respectively. In conclusion, plasma EVs contain distinct protein signatures associated with steatosis, steatohepatitis, and fibrosis in MASLD. These preliminary findings support the potential utility of plasma EVs as non-invasive biomarkers and provide insights into disease pathophysiology. However, further validation in larger, independent cohorts is necessary to confirm these associations and establish their clinical relevance.

## 1. Introduction

Metabolic dysfunction-associated steatotic liver disease (MASLD) currently affects 38% of the global adult population [1]. The burden of MASLD is projected to rise with increasing rates of type 2 diabetes mellitus (T2DM) and obesity worldwide [2]. MASLD encompasses a spectrum ranging from isolated steatosis, defined as fat accumulation in at least 5% of hepatocytes, to metabolic dysfunction-associated steatohepatitis (MASH), fibrosis and ultimately cirrhosis or even liver cancer [3,4]. Often, MASLD remains unnoticed by both patients and physicians since specific symptoms are typically absent until progression into advanced stages of cirrhosis. This underscores the need for improved strategies to detect and stage MASLD early in at-risk individuals.

Currently, liver biopsy remains the reference standard for diagnosing and staging MASLD, allowing for the differentiation between isolated steatosis and MASH and the assessment of fibrosis severity. However, a liver biopsy is invasive [5], labor-intensive, prone to sampling error [6] and subject to significant inter-observer variability [7]. These limitations underscore the critical need for non-invasive diagnostic alternatives. In recent years, several non-invasive tests (NITs) have been proposed for the detection of different stages of disease. Conventional ultrasonography is widely used for the detection of steatosis as it is widely available, well-tolerated and inexpensive [8]. Yet, it has limited sensitivity when <20% of the liver is steatotic [9] and in individuals with morbid obesity [10]. Vibration-controlled transient elastography (VCTE) uses shear wave elastography and reports a controlled attenuation parameter (CAP) and a liver stiffness measurement (LSM) as proxies for liver steatosis and fibrosis, respectively. While VCTE demonstrates good accuracy in the detection of steatosis and fibrosis [11], it cannot identify steatohepatitis and its clinical utility is limited by inter- and intra-operator variability [12] as well as poor availability. Current guidelines recommend a multi-step approach consisting of a blood-based score, such as the fibrosis-4 score (FIB4), followed by imaging techniques, such as VCTE, for the detection of fibrosis in adults with MASLD [13]. Tests of specific collagen-related blood constituents, such as the enhanced liver fibrosis-test (ELF), may serve as alternatives to imaging techniques. Over the years, several quantitative magnetic resonance (MR) techniques have proven accurate to detect different stages of MASLD [8,14]. Proton density fat fraction (PDFF) and magnetic resonance elastography (MRE) can accurately detect hepatic steatosis and fibrosis [15,16], respectively, whilst iron-corrected T1 (cT1) relaxation time has been proposed as a marker for MASH [17]. Although these NITs show promising diagnostic performance, their clinical application can be limited for reasons of scalability. Consequently, there is growing interest in identifying additional, easily accessible circulating biomarkers to enhance current diagnostic efforts.

Extracellular vesicles (EVs) have emerged as promising candidates for biomarker discovery in MASLD [18]. EVs are lipid bilayer-enclosed structures secreted by cells, carrying a diverse range of bioactive cargo, including proteins, lipids, and nucleic acids [19]. EVs are continuously shed or bud from cells, both in normal and stressed conditions, reflecting the physiological or pathological state of the organ from which they originate, including the liver in the case of MASLD. These vesicles facilitate intercellular communication and play a role in key pathological processes of MASLD, such as pro-inflammatory and fibrotic pathways [20]. Detectable in various bodily fluids, including plasma, EVs provide an attractive, minimally invasive platform for disease monitoring and potential therapeutic targeting [19]. The present study aimed to investigate whether plasma EV-derived proteins correlate with different pathological stages of MASLD, assess their relationship with imaging data, and explore their potential as non-invasive biomarkers for diagnosing and staging MASLD.

## 2. Materials and Methods

### 2.1. Study Population

Data from the Amsterdam MASLD-MASH cohort (ANCHOR) study were used. The ANCHOR study is an ongoing prospective longitudinal observational study that aims to identify and validate non-invasive diagnostic methods for the assessment of MASLD [21]. It is conducted at the Amsterdam UMC in compliance with the principles of the Declaration of Helsinki and according to Good Clinical Practice guidelines. The study has been registered in the Dutch Trial Register (registration number NTR7191). Inclusion criteria are hepatic steatosis on abdominal ultrasound or VCTE and age ≥ 18 years. All participants gave written informed consent before inclusion and fasted at least four hours prior to the study procedures. This study has previously been described in detail [21].

### 2.2. Blood Sampling

Blood samples were collected and routine laboratory measurements were analyzed according to local standard protocol. Heparinised plasma (BD, ref tube: 367376) was collected to be stored in a designated biobank to be used for isolation of EVs. Tubes were centrifuged at 1400× *g* for 15 min. After centrifugation, samples were aliquoted and stored in a −80 °C freezer within 3 h of collection to preserve sample integrity. Samples remained frozen at −80 °C until they were used for analyses. As such, all samples underwent one freeze–thaw cycle.

### 2.3. Liver Biopsy

Percutaneous ultrasound-guided liver biopsies were performed by either an interventional radiologist or a hepatologist according to local standard procedures. All histological specimens were tandem read by two expert pathologists who were blinded to all other data. Histological parameters were defined with the use of the steatosis, activity and fibrosis (SAF) score (including lobular inflammation and hepatocyte ballooning), classifying non-MASLD, MASLD without steatohepatitis or MASH [22], and steatosis and fibrosis stage. The degree of steatosis was determined by the percentage of hepatocytes containing large or medium-sized intracytoplasmic lipid droplets, and was scored on a scale from 0 to 3: 0 (<5%), 1 (5–33%), 2 (34–66%), and 3 (>67%). MASH was defined as the presence of steatosis (≥S0) along with the presence of both lobular inflammation (≥1) and hepatocyte ballooning (≥1) [22]. Fibrosis staging was performed according to the MASH Clinical Research Network (CRN) system [23]: stage 0 (F0), no fibrosis; stage 1 (F1), perisinusoidal fibrosis in zone 3 (1a or 1b) or periportal fibrosis (1c); stage 2 (F2), perisinusoidal and periportal fibrosis without bridging; stage 3 (F3), bridging fibrosis; and stage 4 (F4), cirrhosis.

### 2.4. Liver MRI Acquisition and Post-Processing

Participants underwent a single-session liver MRI scan at a 3T MR scanner (Ingenia, Phillips, Best, the Netherlands). A magnitude-based PDFF scan was conducted with a multi-slice multi-echo gradient echo sequence. Prominent vessels, bile ducts, liver edges, and image artifacts were avoided in all post-processing steps. Three circular regions of interest (ROIs) were placed in the liver on three different slices on the first echo time image. A multi-echo, multi-frequency water and fat signal model (including correction for T2* effects) was fitted (Matlab version 2021b; The Mathworks Inc., Natick, MA, USA) to the mean signal intensity in the three ROIs per echo time to obtain PDFF [24]. MRE scans were performed using a fractionally encoded multi-slice gradient echo acquisition (Ristretto) [25]. A gravitational transducer was placed on the midaxillary line at the height of the xiphoid process and had a vibration frequency of 50 Hz [26]. Post-processing of MRE data was performed according to an earlier publication [27]. An ROI in the liver is drawn in the three central slices on the magnitude images. Voxels where the viscoelastic properties could not reliably be determined (nonlinearity > 50%) were excluded from the ROI. The LiverMultiScan^®^ protocol (Perspectum Ltd., Oxford, UK) was used to perform iron-corrected T1 mapping of the liver [28]. The median cT1 value of the whole liver in four slices was calculated.

### 2.5. EV Isolation

EVs were isolated from 1 mL of thawed heparinized plasma samples following a previously established three-step ultracentrifugation protocol, as described in detail in earlier studies [29]. Briefly, samples were diluted with cold phosphate-buffered saline (PBS, 1×) (Gibco, Carlsbad, CA, USA; #10010023) and subjected to sequential centrifugation at 20,000× *g* for 30 min, 110,000× *g* for 2 h, and a final wash at 110,000× *g* for 1 h, all performed at 4 °C using a SW 55 Ti rotor (Beckman Coulter Inc., Brea, CA, USA; #342196). Pellets were resuspended in PBS or lysis buffer for downstream analyses.

### 2.6. Nanoparticle Tracking Analysis

The size distribution and concentration of EVs were assessed using nanoparticle tracking analysis (NTA) with a NanoSight LM14 instrument (Malvern Instruments Ltd., Malvern, UK) equipped with a 404 nm blue laser (70 mW) and an SCMOS camera (Hamamatsu Photonics K.K., Hamamatsu, Japan). Samples were diluted in 1 mL of PBS before measurement. Instrument calibration was performed with 100 nm polystyrene beads, and a blank run was completed before sample analysis. Each sample was measured five times with 60 s videos per measurement. Data were analyzed using NTA software version 3.0, with a threshold setting of 5.

### 2.7. Western Blot Analysis

The presence of EV-specific protein markers was confirmed by Western blotting (File S1). 10 μg protein from random samples was separated on 10% SDS-PAGE gels, followed by semi-dry transfer onto nitrocellulose membranes (Bio-Rad, Hercules, CA, USA). Membranes were blocked with 5% BSA and incubated overnight at 4 °C with the following primary antibodies: CD9 (Cell Signalling Technology, Danvers, MA, USA; #13174S), CD63 (Santa Cruz Biotechnology, Dallas, TX, USA; #15363), CD81 (Invitrogen, Waltham, MA, USA; #10630D), and TSG-101 (Santa Cruz Biotechnology; #7964). After washing, membranes were incubated with HRP-conjugated secondary antibodies: Goat Anti-Rabbit IgG (Agilent Technologies, Santa Clara, CA, USA; #P0448) or Rabbit Anti-Mouse IgG (Agilent Technologies; #P0260). Signal detection was performed using the ChemiDoc XRS system (Bio-Rad).

### 2.8. Mass Spectrometry Analysis

Mass spectrometry procedures were performed following protocols previously established and validated in an earlier study [29]. In brief, EV samples (25 μg total protein) were loaded onto 4–12% Bis-Tris precast gels (Invitrogen; #NP0335BOX) and briefly separated at 100 V for a maximum of 5 min. Gels were stained with Biosafe Coomassie G-250 (Bio-Rad; #1610786) and excised as a single band. Gel pieces were sequentially washed with 70% 100 mM NH_4_HCO_3_/30% acetonitrile, 50% 100 mM NH_4_HCO_3_/50% acetonitrile, and 100% acetonitrile, followed by drying at 37 °C. Reduction and alkylation were performed using 10 mM dithiothreitol (DTT) and 55 mM iodoacetamide (both in 100 mM NH_4_HCO_3_). After further washing and drying, gel pieces were digested overnight at 37 °C with sequencing-grade trypsin (Promega, Madison, WI, USA) at a 1:100 enzyme-to-substrate ratio. Peptides were extracted with 75% acetonitrile/5% formic acid, concentrated using a SpeedVac (Thermo Fisher Scientific, Waltham, MA, USA), and resuspended in 0.1% formic acid for liquid chromatography- mass spectrometer (LC-MS) analysis. Mass spectrometric analysis was conducted using an Orbitrap Exploris 480 mass spectrometer (Thermo Scientific, Wilmington, DE, USA) equipped with a nano-electrospray ion source, coupled to an Evosep One liquid chromatography system (Evosep, Odense, Denmark). Peptides were separated on an EV1137 Performance column (15 cm × 150 μm, 1.5 μm particle size; Evosep) under a 30SPD gradient. Data acquisition was performed in positive ion mode with data-independent acquisition (DIA), a precursor mass range of 400–1000 m/z, and field asymmetric waveform ion mobility spectrometry (FAIMS) settings switching between −45 V and −60 V. Raw data were analyzed using Spectronaut (version 17.5.230413; Biognosys) with direct DIA mode, referencing the human SwissProt database (www.uniprot.org, accessed on 14 February 2024, 20,350 entries).

### 2.9. Statistical Analysis

Baseline characteristics of the study population were summarised as means with standard deviations (±SD), medians with interquartile ranges (IQR) or counts with corresponding percentages (%), as appropriate. Differences in demographic and clinical variables were assessed using independent sample t-tests or Mann–Whitney U-tests for continuous variables, and chi-squared tests for categorical variables, depending on the distribution and type of data. Protein peak area data were normalized on a log_2_ scale and reported as normalized protein expression values, representing relative quantification in arbitrary units. This normalization precluded direct comparisons of absolute levels between different proteins, limiting analyses to comparisons of the same protein across different samples. Proteins with more than 30% missing values across all samples were excluded from further analysis. Contaminant proteins were removed based on the curated FASTA and spectral libraries to ensure high-confidence protein identification [30]. As a result, a total of 370 proteins were retained for downstream analysis.

Intergroup comparisons of plasma EV protein concentrations were performed using the limma package in RStudio version 4.2.1. The differential analysis compared participants with mild (S1) vs. severe (S3) steatosis, those with MASH vs. those with MASLD without steatohepatitis, and those with no to mild (F0-F1) vs. advanced (≥F3) fibrosis. *p*-values were adjusted for multiple testing using the Benjamini–Hochberg procedure. Proteins were considered differentially expressed if they met either of the following criteria: (1) adjusted *p*-value ≤ 0.05; or (2) nominal *p*-value ≤ 0.05 and |log_2_ fold change| ≥ 1. Boxplots were employed to visualize EV protein concentrations per steatosis grade and fibrosis stage. Receiver operating characteristic (ROC) analysis, with the area under the curve (AUC) and corresponding 95% confidence intervals (CI), was used to evaluate the discriminative ability of plasma proteins. Correlation analyses of plasma proteins with MRI-based liver imaging were conducted using Spearman’s rank correlation coefficient. A two-tailed *p*-value ≤ 0.05 was considered statistically significant.

## 3. Results

### 3.1. Participant Characteristics

A total of 70 participants with histologically proven MASLD were included in this study, of whom 47 (67.1%) met the criteria for MASH. Median age was 49.0 (IQR: 37.2–60.2) years and 55.7% of participants were men (Table 1). cT1 values were higher in those with MASH compared to those without steatohepatitis (926.0 (95% CI: 889.0–990.0) vs. 850.0 (95% CI: 786.5–890.5) ms, *p* < 0.001) (Table 1).

**Figure 1:**
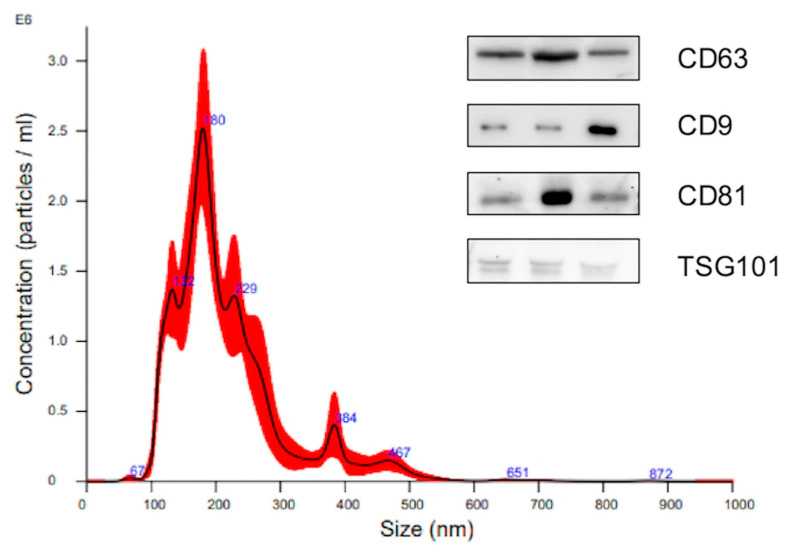
Characterization of plasma extracellular vesicles. Nanoparticle tracking analysis (NTA) showing the size distribution and concentration of isolated plasma EVs, with the majority of particles ranging between 100–200 nm. Western blot (File S1) analysis confirms the expression of EV-enriched markers CD63, CD9, CD81, and TSG101, supporting the identity of the isolated EV population.

NTA confirmed that the diameter of EVs was on average 100–200 nm. Western blot (File S1) showed that CD63, CD9, CD81, and TSG101, the characteristic markers of EVs, were enriched in EVs from plasma (Figure 1).

### 3.2. Plasma EV Proteins as Markers for Steatosis

**Figure 2:**
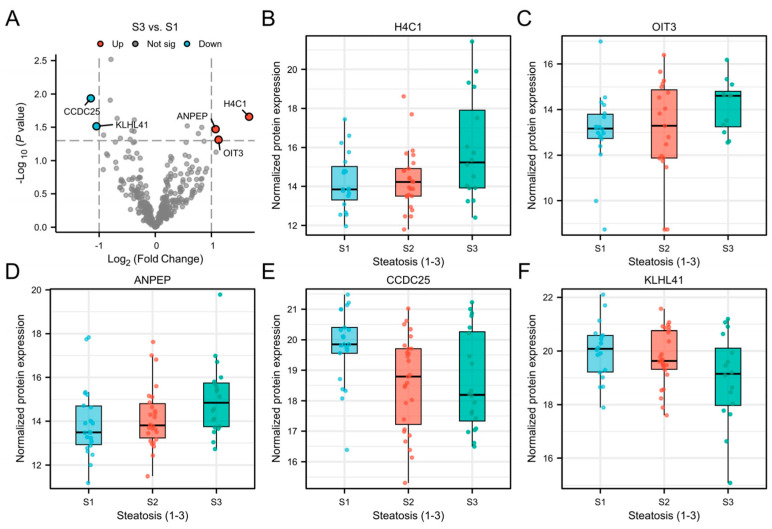
Volcano plot showing differentially abundant plasma EV proteins between participants with S1 vs. S3 (**A**) and boxplots of differentially abundant plasma EV proteins per steatosis grade; H4C1 (**B**), OIT3 (**C**), ANPEP (**D**), CCDC25 (**E**) and KLHL41 (**F**). EV, extracellular vesicles; S1, mild steatosis; S2, moderate steatosis; S3, advanced steatosis; H4C1, histone H4; OIT3, oncoprotein-induced transcript 3; ANPEP, alanyl aminopeptidase; CCDC25, coiled-coil domain-containing protein 25; KLHL41, Kelch-like protein 41.

Plasma EV protein levels were compared between participants with mild steatosis (S1; *n* = 22) and those with severe steatosis (S3; *n* = 20) (Supplementary Table S1). After adjustment for multiple testing, none of the quantified plasma EV proteins were significantly different in abundance (all adjusted *p* > 0.1). However, five EV proteins were nominally significantly different (|log_2_ fold change| > 1, *p* ≤ 0.05) (Figure 2A). Among these proteins, histone H4 (H4C1), oncoprotein-induced transcript 3 (OIT3), and alanyl aminopeptidase (ANPEP) were elevated in participants with S3, whereas coiled-coil domain-containing protein 25 (CCDC25) and Kelch-like protein 41 (KLHL41) were decreased (Figure 2B–F). Further analysis comparing participants with <S3 vs. S3 revealed that H4C1 remained nominally significantly elevated in participants with S3 (log_2_ fold change = 1.62, *p* = 0.004) (Supplementary Figure S1A). Similarly, when comparing participants with <S2 vs. ≥S2, CCDC25 remained nominally significantly downregulated (log_2_ fold change = −1.18, *p* = 0.003) (Supplementary Figure S1B).

Among the individual proteins, CCDC25 had the highest AUC of 0.72 (95% CI: 0.59–0.85) for differentiating <S2 vs. ≥S2 and OIT3 had the highest AUC of 0.69 (95% CI: 0.53–0.85) for differentiating <S3 vs. S3 (Table 2). To further explore the association between EV protein levels and steatosis severity, the nominally significant proteins were correlated with PDFF. Among the five proteins identified, only KLHL41 exhibited a significant correlation with PDFF with a Spearman’s R of −0.34 (*p* = 0.016) (Table 2).

### 3.3. Plasma EV Proteins as Markers for MASH

**Figure 3:**
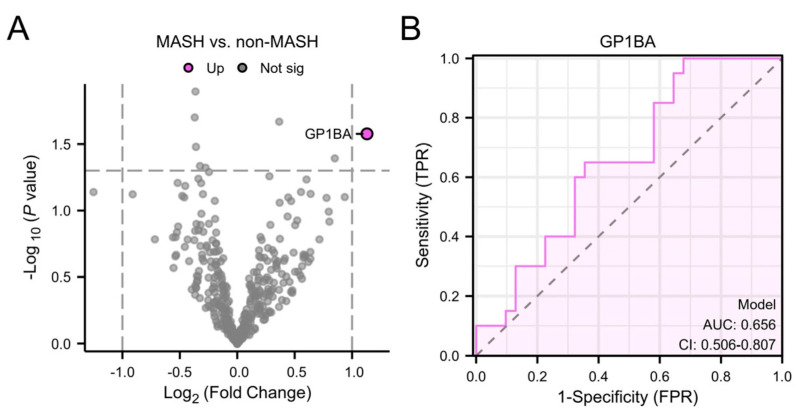
Volcano plot showing differentially abundant plasma EV proteins between participants with MASH and those without steatohepatitis (**A**) and Receiver operating characteristic (ROC)-curve of GP1BA for distinguishing between MASH from MASLD without steatohepatitis (**B**). MASH, metabolic dysfunction-associated steatohepatitis; TPR, true positive rate; AUC, area under the curve; CI, confidence interval; FPR, false positive rate; GP1BA, glycoprotein Ib alpha chain.

Plasma EV protein profiles were compared between participants with MASH (*n* = 47) and those without steatohepatitis (*n* = 23) (Table 1). After adjusting for multiple comparisons, no proteins were found to be significantly differentially abundant (all adjusted *p* > 0.1). Glycoprotein Ib alpha chain (GP1BA) was nominally significant (log_2_ fold change = 1.13, *p* = 0.026), showing elevated expression levels in participants with MASH (Figure 3A). GP1BA had an AUC of 0.66 (95% CI: 0.51–0.81) for the detection of MASH (Figure 3B). Correlation of cT1 and GP1BA had a Spearman’s R of 0.22 (*p* = 0.173).

### 3.4. Plasma EV Proteins as Markers for Fibrosis

**Figure 4:**
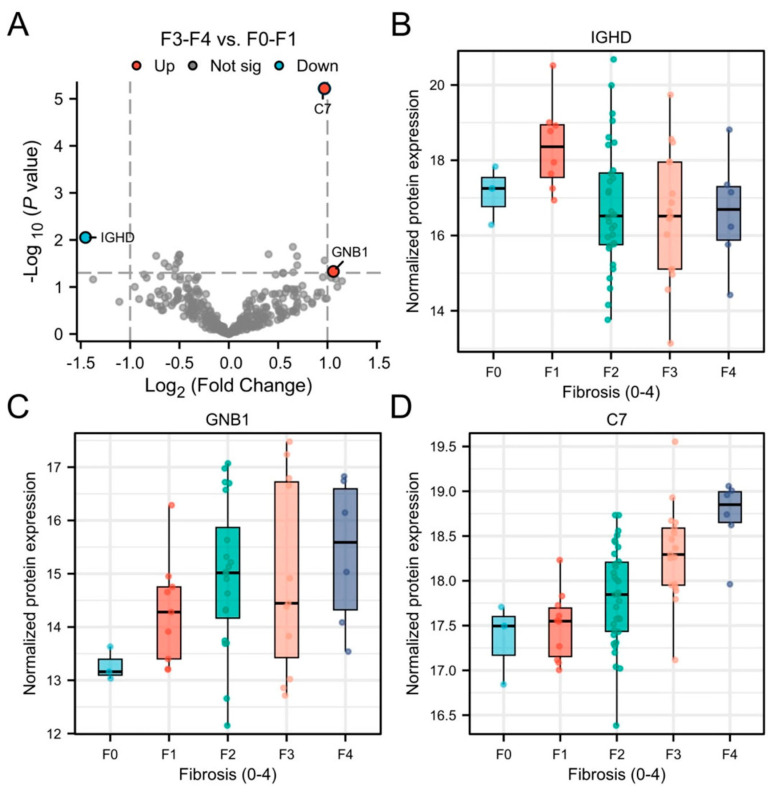
Volcano plot showing differentially abundant plasma EV proteins between participants with F0-1 vs. ≥F3 (**A**) and boxplots of differentially abundant plasma EV proteins per fibrosis grade; IGHD (**B**), GNB1 (**C**) and C7 (**D**). F0, no fibrosis; F1, mild fibrosis; F2, significant fibrosis; F3, advanced fibrosis; F4, cirrhosis; IGHD, immunoglobulin heavy constant delta; GNB1, guanine nucleotide-binding protein subunit beta-1; C7, complement component 7.

Supplementary Table S2 shows participants characteristics of participants with F0-F1 (*n* = 13) and ≥F3 (*n* = 23) fibrosis. After adjusting for multiple comparisons, complement component 7 (C7) remained statistically significant between F0-F1 vs. ≥F3 (log_2_ fold change = 0.95, adjusted *p* = 0.002). Additionally, immunoglobulin heavy constant delta (IGHD) and guanine nucleotide-binding protein subunit beta-1 (GNB1) were identified as nominally significant (|log_2_ fold change| > 1, unadjusted *p* < 0.05; Figure 4A). Notably, IGHD expression decreased with advancing stages of fibrosis (Figure 4B), whereas GNB1 and C7 levels increased (Figure 4C,D).

The AUC of C7 for differentiating <F2 vs. ≥F2 was 0.80 (95% CI: 0.69–0.91) and for distinguishing <F3 vs. ≥F3 was 0.83 (95% CI: 0.72–0.93) (Supplementary Figure S2 and Table S3). In addition, C7 had a Spearman’s R of 0.38 (*p* = 0.004) with MRE-derived elasticity (Table 3).

## 4. Discussion

Here, we performed a comprehensive proteomic analysis of plasma EVs in patients with MASLD and identified distinct protein signatures with key pathological features. Specifically, H4C1, OIT3, ANPEP, CCDC25 and KLHL41 were identified as potential biomarkers for steatosis, GP1BA as a candidate marker for MASH and C7, IGHD and GNB1 as potential biomarkers for fibrosis severity. These findings highlight the potential of EV-derived proteins to serve as non-invasive biomarkers and to support future diagnostic strategies in MASLD.

Our proteomic analysis revealed elevated levels of H4C1, OIT3 and ANPEP and reduced levels of CCDC25 and KLHL41 in EVs from participants with S3 compared to those with S1. Because EVs encapsulate molecular contents reflective of their cell of origin, they provide insight into intracellular processes and disease-specific alterations. Histone H4, a core nucleosome component essential for chromatin remodeling and transcriptional regulation [31,32,33], was nominally elevated in EVs from participants with S3 vs. S1 and S3 vs. <S3. This may reflect hepatocellular stress or apoptosis, contributing to inflammatory signaling in MASLD. Similarly, OIT3, a liver-enriched protein linked to macrophage polarization and hepatocellular carcinoma (HCC) progression [34,35], was also elevated in S3, suggesting enhanced inflammatory responses and immune infiltration in S3. Interestingly, OIT3 was not increased in participants with MASH compared to those without steatohepatitis. ANPEP, which regulates peptide metabolism and extracellular remodeling, was likewise upregulated and may contribute to fibrosis [36]. In contrast, CCDC25 was reduced in participants with S3 vs. S1 and ≥S2 vs. <S2. Given its role in neutrophil extracellular trap (NET) formation and dendritic activation, this downregulation may reflect immune dysregulation in S3 [37]. CCDC25 has also been proposed as a diagnostic and prognostic biomarker for HCC, with reported links to immune cell infiltration and ferroptosis [38]. KLHL41 was similarly decreased in participants with S3 vs. S1 and showed significant, albeit weak-to-moderate, correlation with PDFF. Although primarily known for its role in muscle development, it may participate in cytoskeletal organization or stress responses in hepatocytes [39]. Overall, diagnostic accuracy was moderate with CCDC25 having the highest performance among differentially expressed proteins for distinguishing <S2 vs. ≥S2, yielding an AUC of 0.72.

Plasma EV-derived GP1BA shows potential as a non-invasive biomarker for MASH, although with only modest diagnostic accuracy, with an AUC of 0.66. GP1BA, a core component of the platelet glycoprotein Ib-IX-V complex, is essential for platelet adhesion and activation. Experimental studies have demonstrated that platelet-derived GP1BA promotes intrahepatic platelet accumulation and activation, thereby driving inflammation and fibrogenesis in MASH models [40]. It also mediates interactions between platelets and Kupffer cells, particularly in advanced fibrosis, further amplifying immune cell recruitment and hepatic inflammation [41,42]. Therapeutic blockade of GP1BA with specific antibodies has been shown to ameliorate MASH features, suggesting its potential as a therapeutic target [41,42]. In the present cohort, GP1BA demonstrated only moderate diagnostic accuracy for the detection of MASH and showed no significant correlation with cT1.

C7 emerged as a promising EV-derived biomarker of fibrosis severity in MASLD demonstrating good diagnostic accuracy for distinguishing <F2 vs. ≥F2 (AUC: 0.80) and <F3 vs. ≥F3 (AUC: 0.83), and showing significant, albeit weak-to-moderate, correlation with MRE-derived elastography. As a key component of the terminal complement cascade, C7 contributes to immune-mediated injury and fibrogenesis, processes central to MASLD progression [43,44]. Elevated hepatic and plasma C7 expression has previously been linked to fibrosis stage [45,46], but the present findings extend this by demonstrating selective enrichment of C7 in circulating EVs. Whether EV-derived C7 participates in fibrogenic intercellular communication, tracks disease progression longitudinally and predicts clinical outcomes remains to be determined. The roles of IGHD and GNB1 in EVs during liver fibrogenesis remain less defined. IGHD, the delta heavy chain of immunoglobulin D (IgD), is expressed on B cells and involved in immune regulation, but its role in liver disease is largely unexplored [47]. GNB1, a core mediator of G protein-coupled receptor (GPCR) signaling, may influence fibrogenesis via activation of HSCs [48,49], however, direct evidence of its involvement, particularly through EV-mediated intercellular communication during liver fibrosis progression, is limited and warrants further investigation.

This study has some limitations. The small sample size, single-center design and heterogeneity of the MASLD population highlight the need for external validation in a larger cohort. Although the ANCHOR cohort includes a metabolically challenged subgroup of patients with a high prevalence of overweight and T2DM, closely reflecting the target population of screening and therapeutic interventions, it may not fully capture all patient subpopulations. Furthermore, the use of liver biopsy has several inherent drawbacks, including interobserver variability, change of sampling error [5,6] and categorical classification of histological features, with a potential loss of granularity. To address this, we also performed MRI. The correlation between plasma EV biomarker levels and imaging parameters (PDFF, cT1, MRE) was absent to weak, likely reflecting the imperfect concordance between histological and imaging assessments of liver pathology. These methods should therefore be viewed as complementary rather than interchangeable as each provides unique but inherently limited insights into disease processes. In addition, although the proteomic profiling revealed potential EV-associated proteins linked to steatosis and fibrosis, most observed differences were modest and did not remain statistically significant after multiple-comparison correction. This likely reflects both the small sample size and shared biological mechanisms among groups (different degrees of steatosis or fibrosis) within the MASLD continuum. No direct mechanistic or technical validation (e.g., ELISA or Western blot) was performed in this study due to limited sample volume, and this will be an important focus of future work. Additionally, it is challenging to obtain pure EV preparations from plasma due to the overlapping size and density profiles of EVs and other plasma components, which may introduce contaminating proteins and lipoproteins into the EV fraction. Moreover, the cellular origins of circulating EVs were not determined, and future studies incorporating cell-type-specific EV analysis or molecular tracing approaches will be needed to elucidate their source and functional relevance. Overall, this work should be regarded as preliminary and exploratory. Larger, multi-center validation studies and mechanistic investigations will be essential to confirm these findings and further clarify the biological and clinical utility of plasma EV proteins in MASLD.

## 5. Conclusions

In summary, our study provides novel insights into the EV proteomic landscape of MASLD, identifying distinct EV-derived protein signatures linked to key pathological features, including steatosis and fibrosis, and to a lesser extent, MASH. These findings highlight the potential of plasma EV-derived proteins as non-invasive biomarkers for disease stratification and underscore the value of EV-based approaches in advancing the understanding of MASLD pathogenesis. However, given the exploratory nature of this work, future studies in larger, independent cohorts are needed to validate these biomarkers and clarify their mechanistic roles.

## Figures and Tables

**Table 1:** Participant characteristics of all participants (*n* = 70) and stratified for those with MASLD without steatohepatitis (*n* = 23) and those with MASH (*n* = 47) and their associated *p*-value.

		All Participants (*n* = 70)	MASLD Without Steatohepatitis (*n* = 23)	MASH (*n* = 47)	*p*-Value
Age, years		49.0 (37.2–60.2)	49.0 (38.5–55.5)	50.0 (37.5–61.0)	0.754 *
Sex	Men, *n* (%)	39 (55.7)	13 (56.5)	26 (55.3)	0.924 **
	Women, *n* (%)	31 (44.3)	10 (43.5)	21 (44.7)	
BMI, kg/m^2^		32.8 (29.5–36.2)	32.0 (29.8–35.4)	33.0 (29.4–36.3)	0.408 *
Waist circumference, cm		112.6 (±13.2)	109.6 (±10.8)	114.1 (±14.1)	0.179 ***
Hip circumference, cm		112.7 (±11.8)	111.2 (±9.8)	113.4 (±12.7)	0.477 ***
T2DM	Yes, *n* (%)	29 (41.4)	6 (26.1)	23 (48.9)	0.068 **
	No, *n* (%)	41 (58.6)	17 (73.9)	24 (51.1)	
Hypertension	Yes, *n* (%)	27 (38.6)	6 (26.1)	21 (44.7)	0.133 **
	No, *n* (%)	43 (61.4)	17 (73.9)	26 (55.3)	
Total bilirubin, µmol/L		9.0 (6.8–12.0)	8.0 (6.5–11.0)	9.0 (7.0–12.0)	0.495 *
ALP, U/L		87.5 (70.0–104.2)	86.0 (68.0–104.0)	88.0 (71.0–105.0)	0.683 *
γGT, U/L		66.0 (36.5–94.0)	66.0 (39.0–95.0)	64.0 (36.5–91.8)	0.817 *
AST, U/L		42.0 (35.0–57.0)	37.0 (29.0–41.2)	50.0 (38.0–74.8)	<0.001 *
ALT, U/L		62.0 (48.0–82.0)	60.0 (48.0–74.5)	62.0 (47.5–107.0)	0.269 *
Steatosis grade	1, *n* (%)	22 (31.4)	14 (60.9)	8 (17.0)	<0.001 **
	2, *n* (%)	28 (40.0)	6 (26.1)	22 (46.8)	
	3, *n* (%)	20 (28.6)	3 (13.0)	17 (36.2)	
Fibrosis stage	0, *n* (%)	3 (4.3)	3 (13.0)	0 (0.0)	<0.001 **
	1, *n* (%)	10 (14.3)	9 (39.1)	1 (2.1)	
	2, *n* (%)	34 (48.6)	10 (43.5)	24 (51.1)	
	3, *n* (%)	17 (24.3)	1 (4.3)	16 (34.0)	
	4, *n* (%)	6 (8.6)	0 (0.0)	6 (12.8)	
PDFF, %		17.7 (14.6–24.8)	17.2 (8.4–22.6)	20.2 (15.4–25.0)	0.158 *
cT1, ms		901.0 (846.0–974.0)	850.0 (786.5–890.5)	926.0 (889.0–990.0)	<0.001 *
MRE, kPa		1.8 (1.7–2.0)	1.7 (1.5–1.9)	1.9 (1.7–2.2)	0.034 *

MASLD, metabolic dysfunction-associated steatotic liver disease; MASH, metabolic dysfunction-associated steatohepatitis; BMI, body mass index; T2DM, type 2 diabetes mellitus; ALP, alkaline phosphatase; γGT, gamma-glutamyl transferase; AST, aspartate aminotransferase; ALT, alanine aminotransferase; PDFF, proton density fat fraction; cT1, iron-corrected T1 mapping; MRE, magnetic resonance elastography. * Mann–Whitney U-test; ** Chi-square test; *** Unpaired *t*-test.

**Table 2:** Diagnostic performance of differentially expressed proteins for steatosis grades and their correlation with PDFF.

Protein	AUC (95% CI)		Correlation with PDFF	
	<S2 vs. ≥S2	<S3 vs. ≥S3	Spearman’s R	*p*-Value
H4C1	0.589 (0.433–0.746)	0.679 (0.511–0.847)	−0.01	0.953
OIT3	0.602 (0.439–0.765)	0.689 (0.527–0.851)	0.08	0.586
ANPEP	0.631 (0.481–0.781)	0.688 (0.551–0.824)	0.16	0.232
CCDC25	0.723 (0.594–0.851)	0.590 (0.427–0.751)	−0.04	0.765
KLHL41	0.606 (0.452–0.761)	0.658 (0.479–0.836)	−0.34	0.016

AUC, area under the curve; CI, confidence interval; PDFF, proton density fat fraction S2, moderate steatosis; S3, advanced steatosis; H4C1, histone H4; OIT3, oncoprotein-induced transcript 3; ANPEP, alanyl aminopeptidase; CCDC25, coiled-coil domain-containing protein 25; KLHL41, Kelch-like protein 41.

**Table 3:** Diagnostic performance of differentially expressed proteins for fibrosis stages and their correlation with MRE-derived elasticity.

Protein	AUC (95% CI)		Correlation with MRE-Derived Elasticity	
	<F2 vs. ≥F2	<F3 vs. ≥F3	Spearman’s R	*p*-Value
C7	0.802 (0.689–0.914)	0.827 (0.723–0.931)	0.38	0.004
IGHD	0.757 (0.629–0.885)	0.582 (0.436–0.729)	−0.06	0.782
GNB1	0.712 (0.560–0.864)	0.577 (0.393–0.761)	−0.04	0.746

AUC, area under the curve; CI, confidence interval; MRE, magnetic resonance elastography; F2, significant fibrosis; advanced fibrosis; F3, advanced fibrosis; C7, complement component 7; IGHD, immunoglobulin heavy constant delta; GNB1, guanine nucleotide-binding protein subunit beta-1.

## Data Availability

Data available on request from the authors.

## Supplementary Materials

The following supporting information can be downloaded at: https://www.mdpi.com/article/10.3390/biom15111596/s1, Table S1. Comparison of demographic and clinical characteristics between S1 and S3 participants. Table S2. Comparison of demographic and clinical characteristics between F0-F1 and F3-F4 participants. Figure S1. (A) Comparison of S3 vs. S1-S2 and (B) Comparison of S2-S3 vs. S1, with proteins identified as upregulated (red), downregulated (blue), or not significant (grey). Key proteins are annotated. Figure S2. Receiver operating characteristic (ROC) curves of plasma extracellular vesicle (EV)-derived C7 for distinguishing patients by fibrosis stage. File S1. Original Western blots.

## Abbreviations

The following abbreviations are used in this manuscript:

ANCHOR	Amsterdam MASLD-MASH cohort
ANPEP	Alanyl aminopeptidase
AUC	Area under the curve
C7	Complement component 7
CAP	Controlled attenuation parameter
CCDC25	Coiled-coil domain-containing protein 25
CI CRN	Confidence interval Clinical research network
cT1 DIA DTT	Iron-corrected T1 mapping Data-independent acquisition Dithiothreitol
ELF	Enhanced liver fibrosis-test
EV FAIMS	Extracellular vesicle Field asymmetric waveform ion mobility spectrometry
F0	No fibrosis
F1	Mild fibrosis
F2	Significant fibrosis
F3	Advanced fibrosis
F4	Cirrhosis
FIB4	Fibrosis-4 score
GNB1	Guanine nucleotide-binding protein subunit beta-1
GP1BA	Glycoprotein Ib alpha chain
H4C1	Histone H4
HCC	Hepatocellular carcinoma
IGHD	Immunoglobulin heavy constant delta
IQR	Interquartile range
KLHL41 LC-MS	Kelch-like protein 41 Liquid chromatography- mass spectrometer
LSM	Liver stiffness measurement
MASH	Metabolic dysfunction-associated steatohepatitis
MASLD	Metabolic dysfunction-associated steatotic liver disease
MR	Magnetic resonance
MRE	Magnetic resonance elastography
NIT	Non-invasive test
OIT3	Oncoprotein-induced transcript 3
PDFF	Proton density fat fraction
ROC	Receiver operating characteristic
ROI	Region of interest
S1	Mild steatosis
S2	Moderate steatosis
S3 SAF	Advanced steatosis Steatosis, activity and fibrosis
SD	Standard deviation
T2DM	Type 2 diabetes mellitus
VCTE	Vibration-controlled transient elastography
