# Supplementary material for: Plasma Extracellular Vesicles Contain Protein Biomarkers for Capturing Stages of Metabolic Dysfunction-Associated Steatotic Liver Disease: A Preliminary Exploratory Study
Source: Biomolecules. 2025 Nov 14;15(11):1596. doi: 10.3390/biom15111596 (PMC12650337; doi:10.3390/biom15111596)
Supplement: Supplementary file 1 [file biomolecules-15-01596-s001.zip › Supplementary figures and tables.pdf]

Supplementary Table S1 Comparison of demographic and clinical characteristics between S1 and S3 participants

	**S1 (*n* = 22)**	**S3 (*n* = 20)**	**p-value**
Age, years	52.5 (47.2, 60.8)	51.0 (34.5, 61.2)	0.51
Sex			0.55
Male	13 (59.1%)	10 (50%)	
Female	9 (40.9%)	10 (50%)	
BMI, kg/m^2	33.4 (30.0, 36.3)	32.8 (29.3, 37.3)	1.0
Waist circumference, cm	115.3 ± 14.0	114.1 ± 14.1	0.79
Hip circumference, cm	114.9 ± 11.6	114.5 ± 11.8	0.90
Type 2 diabetes mellitus	11 (50%)	6 (30%)	0.19
Hypertension	9 (40.9%)	9 (45%)	0.79
Total bilirubin, μmol/L	9.0 (7.0, 12.8)	9.0 (7.0, 11.0)	0.73
ALP, U/L	86.5 (70.2, 96.2)	104.0 (74.0, 120.5)	0.21
GGT, U/L	53.5 (29.2, 115.0)	82.5 (66.2, 130.0)	0.026
AST, U/L	38.0 (29.0, 47.0)	57.0 (38.2, 99.2)	0.006
ALT, U/L	58.0 (42.0, 67.2)	82.0 (55.8, 123.2)	0.017
PDFF, %	13.9 (7.2, 17.5)	24.9 (19.2, 27.8)	< 0.001

Data are presented as means ± standard deviations (SD), medians with interquartile ranges (IQR), or counts with corresponding percentages (%), as appropriate. P-values ≤0.05 were considered statistically significant. MASH, metabolic dysfunction-associated steatohepatitis; BMI, body mass index; ALP, alkaline phosphatase; GGT, gamma-glutamyl transferase; AST, aspartate aminotransferase; ALT, alanine aminotransferase; PDFF, proton density fat fraction.

Supplementary Table S2 Comparison of demographic and clinical characteristics between F0-F1 and F3-F4 participants

	**F0-F1 (*n* = 13)**	**F3-F4 (*n* = 23)**	**p-value**
Age, years	44.0 (33.0, 52.0)	54.0 (43.5, 61.5)	0.07
Sex			0.55
Male	6 (46.2%)	13 (56.5%)	
Female	7 (53.8%)	10 (43.5%)	
BMI, kg/m^2	30.1 (28.4, 33.2)	33.8 (29.9, 36.5)	0.05
Waist circumference, cm	106.0 ± 10.8	117.2 ± 16.1	0.033
Hip circumference,	109.8 ± 11.4	115.0 ± 14.6	0.28

cm			
Type 2 diabetes mellitus	2 (15.4%)	16 (69.6%)	0.002
Hypertension	3 (23.1%)	14 (60.9%)	0.029
Total bilirubin, μmol/L	8.0 (6.0, 10.0)	9.5 (8.2, 13.2)	0.19
ALP, U/L	89.0 (59.0, 108.0)	87.5 (74.0, 106.0)	0.72
GGT, U/L	42.0 (27.0, 96.0)	68.0 (35.0, 129.0)	0.57
AST, U/L	32.0 (27.0, 38.0)	51.0 (37.0, 73.0)	0.006
ALT, U/L	65.0 (48.0, 73.0)	61.0 (40.0, 100.0)	0.78
MRE, kPa	1.6 (1.5, 1.8)	2.2 (1.7, 2.9)	0.004

Data are presented as means ± standard deviations (SD), medians with interquartile ranges (IQR), or counts with corresponding percentages (%), as appropriate. P-values ≤0.05 were considered statistically significant. MASH, metabolic dysfunction-associated steatohepatitis; BMI, body mass index; ALP, alkaline phosphatase; GGT, gamma-glutamyl transferase; AST, aspartate aminotransferase; ALT, alanine aminotransferase; MRE, magnetic resonance elastography.

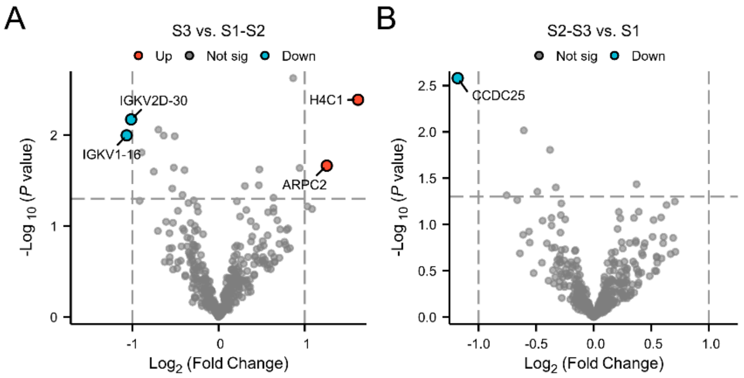

Supplementary Figure S1 (A) Comparison of S3 vs. S1-S2 and (B) Comparison of S2-S3 vs. S1, with proteins identified as upregulated (red), downregulated (blue), or not significant (grey). Key proteins are annotated.

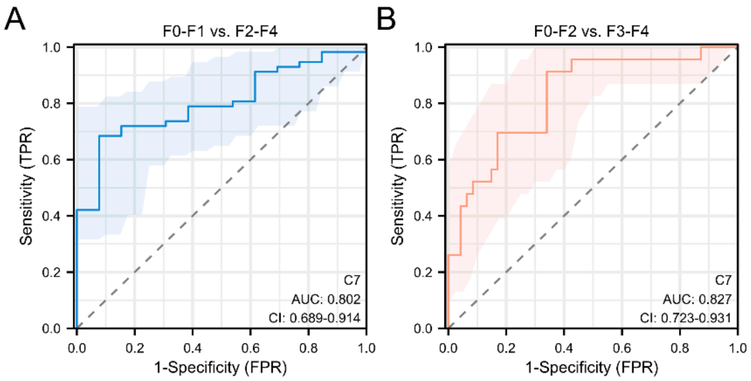

Supplementary Figure S2 Receiver operating characteristic (ROC) curves of

plasma extracellular vesicle (EV)-derived C7 for distinguishing patients by fibrosis stage. (A) F0–F1 vs. F2–F4, with an AUC of 0.802 (95% CI: 0.689–0.914). (B) F0–F2 vs. F3–F4, with an AUC of 0.827 (95% CI: 0.723–0.931).
