# Supplementary figures and images for: Plasma Extracellular Vesicles Contain Protein Biomarkers for Capturing Stages of Metabolic Dysfunction-Associated Steatotic Liver Disease: A Preliminary Exploratory Study
Source: Biomolecules. 2025 Nov 14;15(11):1596. doi: 10.3390/biom15111596 (PMC12650337; doi:10.3390/biom15111596)

Run 1

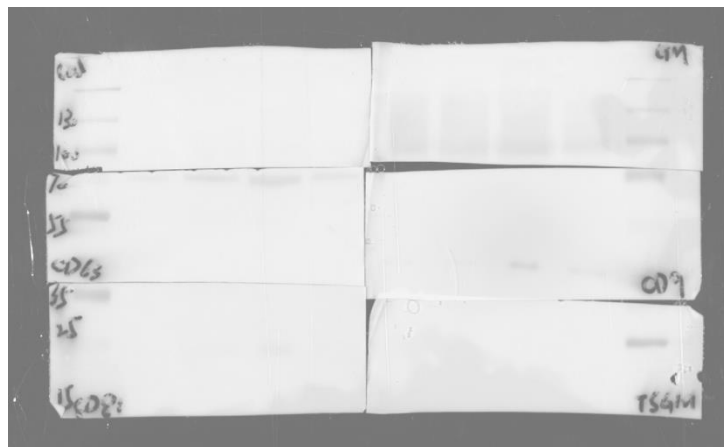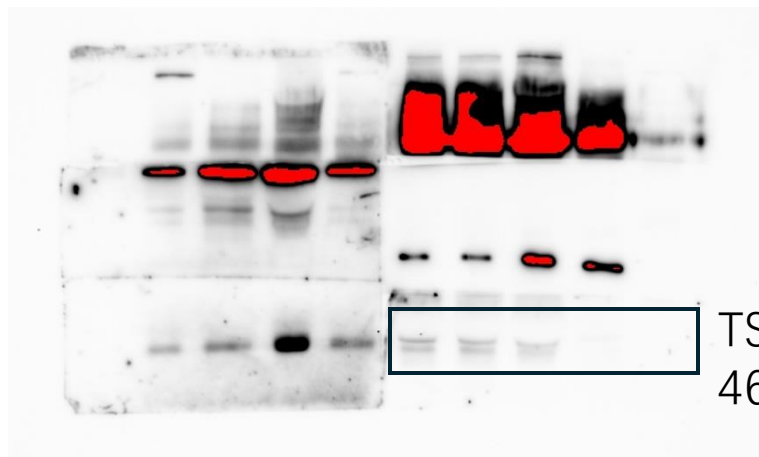

TSG101  
46KD

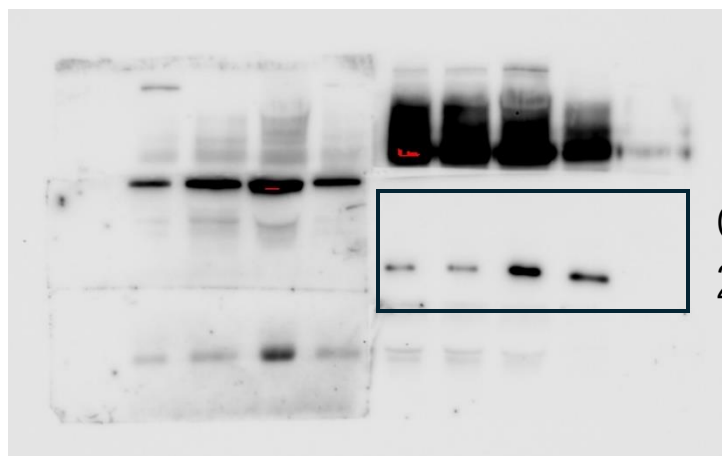

CD9  
24-27KD

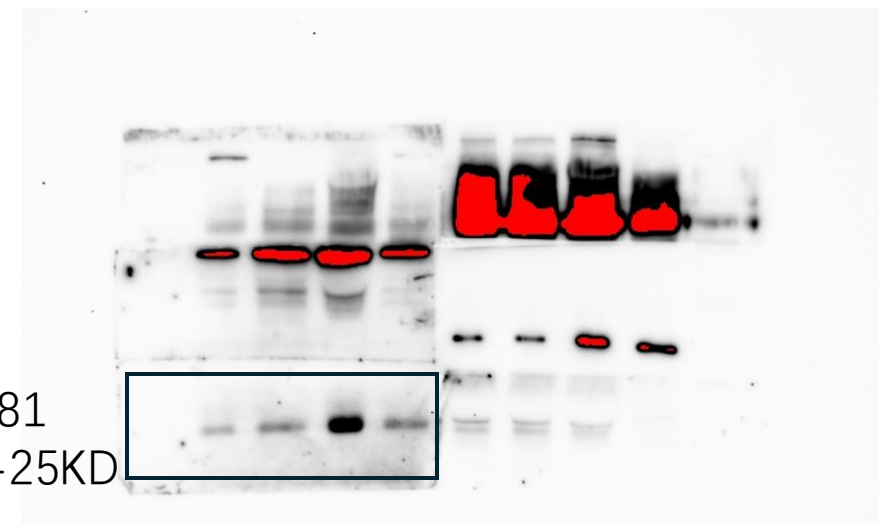

CD81  
20-25KD

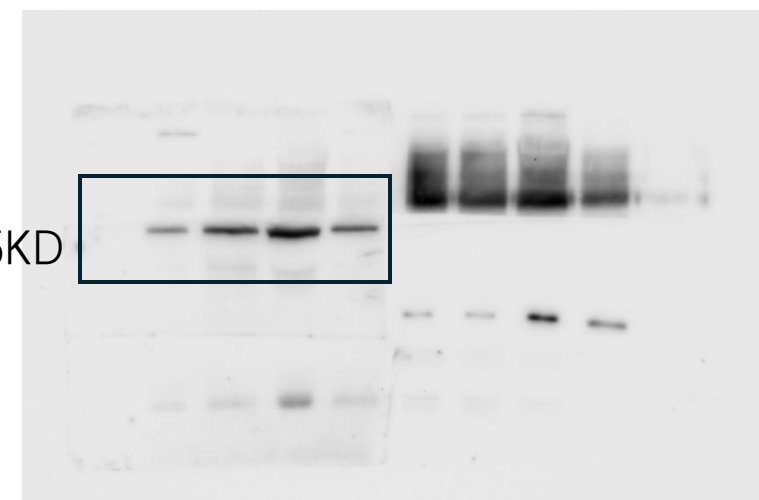

CD63  
50-55KD

Run 2

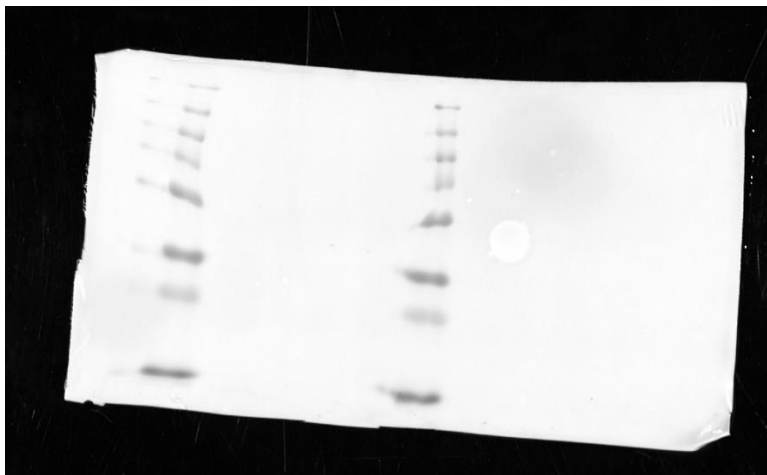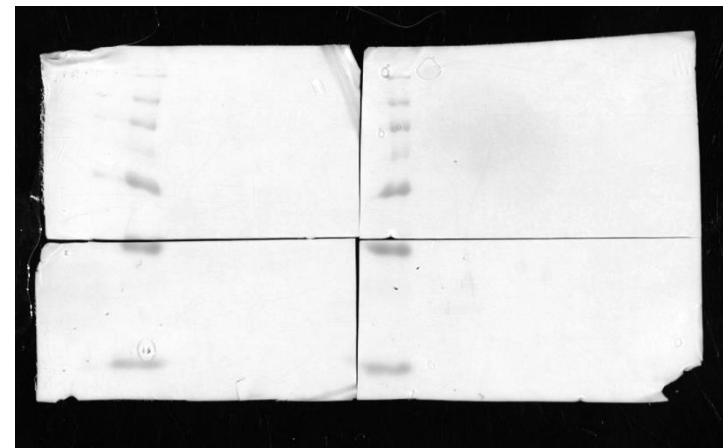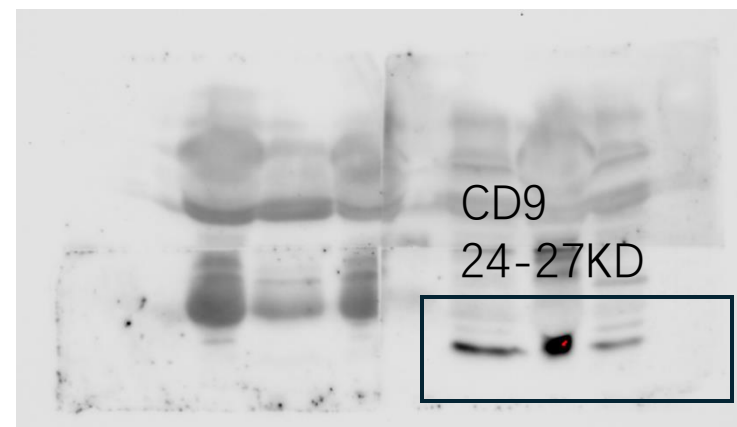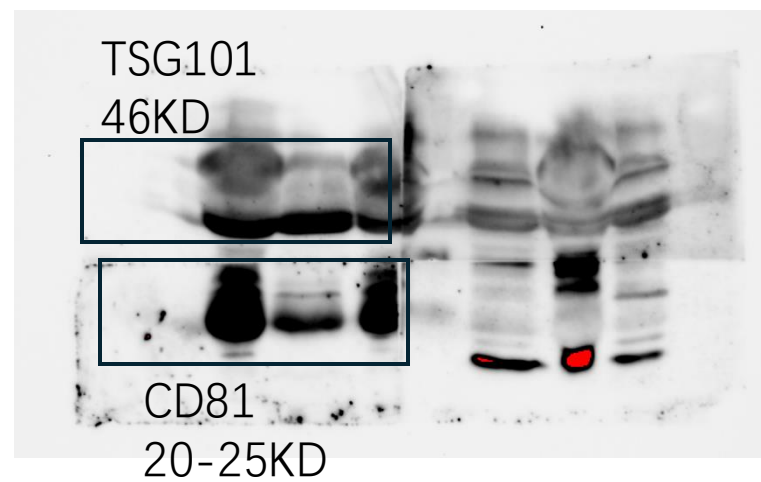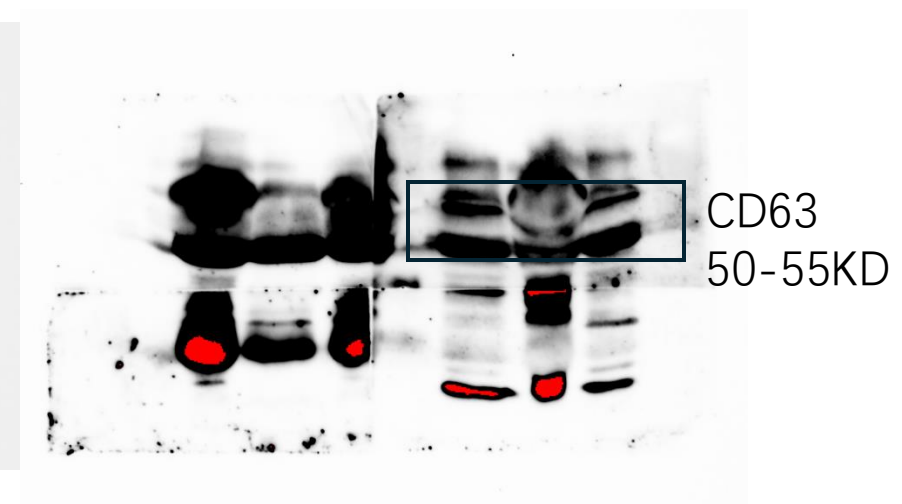

Run 3

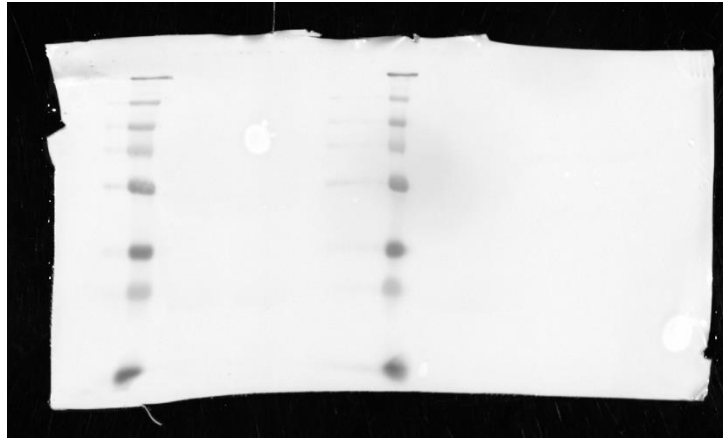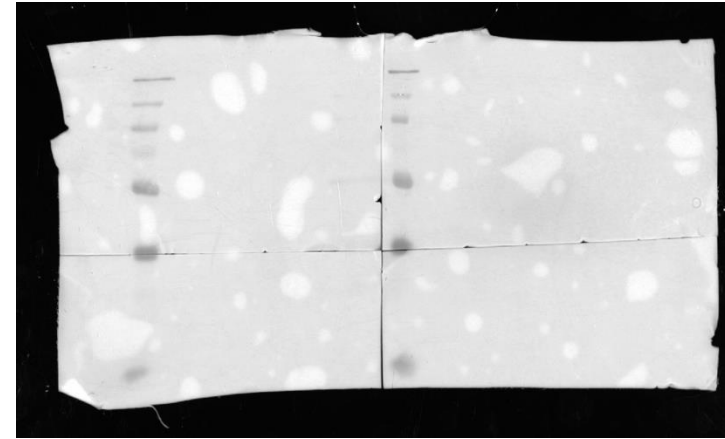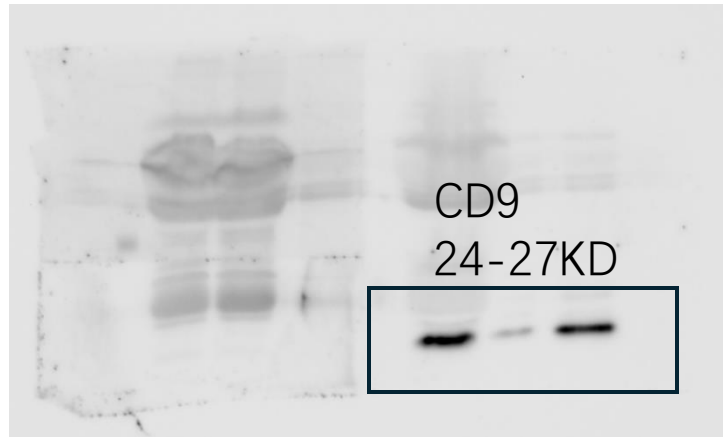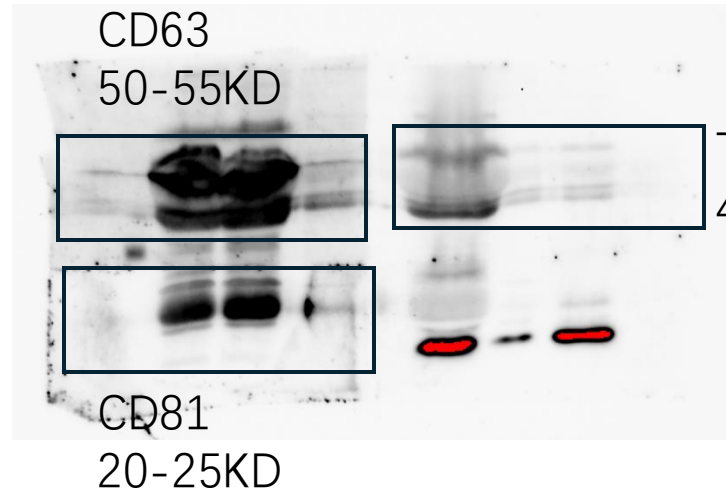

Supplement: Supplementary file 1 [file biomolecules-15-01596-s001.zip › File S1. Original Western blots.pdf]
